# Complete mitochondrial genome of summer flounder *Paralichthys dentatus* (Pleuronectiformes, Paralichthyidae)

**DOI:** 10.1080/23802359.2016.1258340

**Published:** 2016-11-22

**Authors:** Yongjiang Xu, Xuezhou Liu, Bao Shi, Bin Wang

**Affiliations:** a Key Laboratory for the Sustainable Development of Marine Fisheries Ministry of Agriculture, Yellow Sea Fisheries Research Institute Chinese Academy of Fishery Sciences, Qingdao, China;; b Laboratory for Marine Fisheries and Aquaculture, Qingdao National Laboratory for Marine Science and Technology, Qingdao, China

**Keywords:** *Paralichthys dentatus*, mitochondrial genome, genome organization, phylogenetic analysis

## Abstract

We determined the complete nucleotide sequence of the mitochondrial genome for the summer flounder, *Paralichthys dentatus* (Pleuronectiformes, Paralichthyidae). This mitochondrial genome, consisting of 17,033 base pairs (bp), encoded genes for 13 protein-coding genes, 2 ribosomal RNAs, 22 transfer RNAs, and a putative D-loop region like those found in other vertebrates, with the gene order identical to that of typical vertebrates. Phylogenetic analysis showed that *P. dentatus* clustered into one clade with its Paralichthyidae counterparts, *Paralichtchys lethostigma* and *Paralichtchys olivaceus*, which showed the close relationship among these species.

Summer flounder, *Paralichthys dentatus*, is an important commercially and recreationally caught flatfish occurring in continental shelf waters from Maine to Florida in the United States (Morse 1981). In 2002, summer flounder was introduced into China for its relative rapid growth rate and high-temperature tolerance in comparison with native flounder species, *P. olivaceus* (Wang et al. 2004). Recently, hybridization between *P. dentatus* and *P. olivaceus* has been achieved (Tian et al. 2006; Liu et al. 2015). As an alien species, it is essential to investigate the genetic relationship between *P. dentatus* and native species and evaluate gene flow and possible genetic invasion risk. Mitochondrial DNA is a useful marker for analyses of gene flow, hybridization, and introgression for its compactness, maternal inheritance, fast evolutionary rate, and the resulting short coalescence time (Miya et al. 2003; Oh et al. 2007). However, the mitochondrial genome of *P. dentatus* remains unclear.

Ten individuals of *P. dentatus* were sampled from the Great Bay Aquaculture LLC. located at Portsmouth, NH, USA (43˚05′ N, 70˚ 47′ W), the samples were transferred to and stored at our lab located at Yellow Sea Fisheries Research Institute, Qingdao, China. The whole genomic DNA was extracted from muscle tissues and the mitogenome was amplified with universal metazoan primer pairs and completely sequenced with a 3730 × l DNA Analyzer. The complete mitochondrial genome of *P. dentatus* was 17,033 base pairs (bp) in length (GenBank accession no.: KU053334.1), it contained 13 protein-coding genes (PCGs), 2 ribosomal (r)RNA genes (12S rRNA, 16S rRNA), 22 transfer (t) RNA genes, and a putative control region (D-loop) as do most other fish mitogenomes (Boore 1999; Miya et al. 2003). Overall base compositions of the *P. dentatus* mitochondrial genome were as follows: A, 28.15% (4795/17,033); C, 27.96% (4763/17,033); G, 16.90% (2878/17,033); and T, 26.99% (4597/17,033). 12 PGCs of *P. dentatus* had the typical mitochondrial start codon ATG, while the *COI* had a GTG start codon. Six PCGs (*ND1*, *COI, ATPase 8*, *ND4L*, *ND5*, and *ND6*) ended with complete termination codons (TAA), while the other seven used the incomplete stop codon TA– (*ND2*, *ATPase 6*, and *COIII*) or T–– (*COII*, *ND3*, *ND4*, and *CytB*). Three reading-frame overlaps occurred on the same strand including ATPase subunit (ATPase) 8 and 6 overlapped by 10 nucleotides, *ND4L* and *ND4* overlapped by seven nucleotides, and *ND5* and *ND6* overlapped by four nucleotides. The tRNA genes ranged from 65 to 74 bp and were distributed among the genome. The 12S rRNA and 16S rRNA genes were 948 and 1712 nucleotides long, respectively, and were located between *tRNA^Phe^* and *tRNA^Leu^*(UAA), separated by *tRNA^Val^.* The control D-loop region in *P. dentatus* was located between *tRNA^Phe^* and *tRNA^Pro^*, which was 1338 bp in length, this control region was AT-rich, with an AT content of 61.84%. The phylogenetic tree between *P. dentatus* mitochondrial genome and other 13 Pleuronectiformes fishes showed that Paralichthyidae fishes clustered into one branch with Pleuronectidae fishes, and the Paralichthyidae species including *P. dentatus*, *P. lethostigma*, and *P. olivaceus* clustered into one sub-branch with a bootstrap value of 100% (Figure 1).

**Figure 1.**
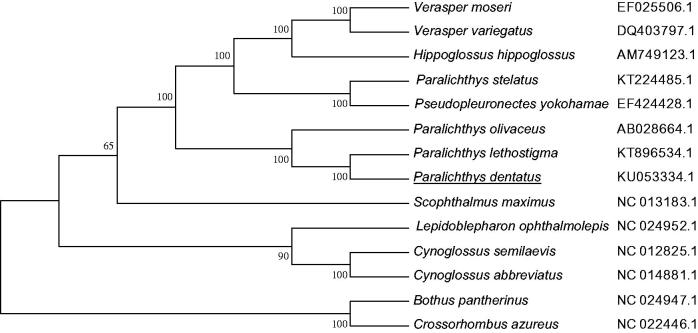
Phylogenetic tree of NJ analyses based on complete mitochondrial amino acid sequences of *P. dentatus*. The species studied in this work is underlined.
